# Traumatic Posterior Urethral Strictures in Children and Adolescents

**DOI:** 10.3389/fped.2019.00024

**Published:** 2019-02-19

**Authors:** Miguel Podesta, Miguel Podesta

**Affiliations:** Urology Unit, Department of Surgery, Hospital de Niños Ricardo Gutierrez, associated to the University of Buenos Aires, Buenos Aires, Argentina

**Keywords:** urethra, injuries, urethral stricture, surgery, children, adolescents

## Abstract

**Background:** Management of partial or complete traumatic urethral disruptions of the posterior urethra in children and adolescents, secondary to pelvic fracture poses a challenge. Controversy exists as to the correct acute treatment of posterior urethral injuries and delayed management of PFPUDDs. We reviewed the urological literature related to the treatment of traumatic posterior urethral injuries and delayed repair of these distraction defects in children and adolescents.

**Material and Methods:** There are few long-term outcomes studies of patients who underwent PFPUDDs repairs in childhood; most reports included few cases with short follow up. We excluded studies in which the cohort of patients was heterogeneous in terms of stricture disease, etiology and location.

**Results:** Primary cystostomy and delayed urethroplasty is the traditional management for PFPUIs. Immediate repair is rarely possible to perform. Realignment of posterior urethral rupture in children is indicated in special situations: (a) concomitant bladder neck tears, (b) associated rectal lacerations, (c) long disruptions of the urethral ends. Before delayed reconstruction ascending urethrography and micturating cystourethrogram along with retrograde and antegrade urethroscopy define site and length of the urethral gap. However, the most accurate evaluation of the characteristics of the distraction defect is made when surgical exposure reveals the complexity of the ruptured urethra. Partial ruptures may be managed with urethral stenting or suprapubic cystostomy, which may result in a patent urethra or a short stricture treated by optical urethrotomy. The gold standard treatment for PFPUDDs in children is deferred excision of pelvic fibrosis and bulbo-prostatic tension-free anastomosis, provided a healthy anterior urethra is present. Timing of delayed repair is at 3 to 4 months after trauma. Some urologists prefer either the perineal access or the transpubic approach to restore urethral continuity in children with PFPUDDs. Substitution urethroplasties are used in children with PFPUDDs, when anastomotic repair can't be achieved due to severe damage of the bulbar urethra.

**Conclusion:** As evidenced in this review the progressive perineo-abdominal partial transpubic anastomotic repair has advantages over the isolated perineal anastomotic approach in patients with “complex” PFPUDD. This approach provides wider exposure and facilitates reconstruction of long or complicated posterior urethral distraction defects

## Introduction

Trauma is a major cause of morbidity and mortality in children (1). Urethral trauma producing stricture disease in pediatric patients most often results from fracture of the pelvis, straddle injuries or iatrogenic urethral manipulation (2). The incidence of pediatric PFUIs has been estimated to be between 1 and 5%; however, Tarman et al. reported in a series of 212 children with fracture of the pelvis that the occurrence of urethral lesions was <1% (3, 4). In childhood, the majority of PFPUIs occur as pedestrians hit by a motor vehicle rather than as passengers of a vehicle involved in an accident (5–7).

A distinction should be made between the terms urethral stricture and rupture of the urethra. The former is a narrowing of the urethral canal as a result of changes in its walls caused either by inflammation or by trauma, while the latter results in partial or complete separation of the injured urethral extremities, followed by the development of dense fibrous tissue between the torn urethral ends (8). Posterior urethral strictures are generally the consequence of endoscopic trauma at the site of the sphincter active membranous urethra, but urethral continuity is preserved. In adults, PFPUIs usually involve the membranous urethra at some point between the apex of the prostate and the posterior bulbar urethra, and commonly result in a short urethral distraction defect associated with localized pelvic fibrosis (9, 10). Turner Warwick introduced the term “complex” posterior urethral distraction defect due to a pelvic fracture (PFPUDD) when one or more of the following features are present: (a) the distraction defect length is long (≥3 cm) surrounded by extensive pelvic fibrosis and (b) it is accompanied by para-urethral diverticula, false passages, fistulas, rectal tears or simultaneous bladder neck lesion. These complex urethral distraction defects require a wider surgical exposure to restore urethral continuity and to correct associated adjacent traumatized structures (8).

Although PFPUDD pathogenesis in children tends to follow a similar pattern to the one in adults, several key elements require consideration. Some authors have inferred that the location of the traumatic urethral injury in children is less predictable due to the abdominal position of the bladder and immaturity of the prostate (11). Further factors to consider in pediatric patients include: (a) urethral distraction defects tend to be longer than in adults because of marked upwards displacement of the bladder and prostate, (b) double injuries at the bladder neck and the membranous urethra are more frequently observed and (c) pre-pubertal perineum size may make it difficult to reach a high lying proximal urethral end (12–14).

We reviewed previous manuscripts by searching PubMed Medline electronic database for clinically relevant articles. There are not many long-term outcomes studies of patients who underwent PFPUDDs repairs in childhood; most published reports have included few cases with short follow-up. We excluded studies in which the pediatric patients were heterogeneous in terms of stricture disease etiology and location. Thus, the objective of this review will focus on the urological literature related to the acute management of traumatic injuries of the posterior urethra and delayed repair of posttraumatic obliterate posterior distraction defects in children and adolescents.

## Materials and Methods

Patients who suffer from severe pelvic injury are usually in shock and urinary symptoms are often overlooked. At that moment, clinical differentiation between an extra-peritoneal bladder lesion and a posterior urethral rupture is difficult; however, if a distended bladder is palpated, the rupture might be urethral. Upon suspicion of urethral injury in any boy who has had a pelvic fracture, appropriate evaluation is mandatory when the clinical condition of the patient is stable. Urethral catheterization is to be avoided in order not to aggravate the urethral injury or introduce infection to the pelvic hematoma (6, 8). Retrograde urethrography in the oblique position is the most effective examination to diagnose a urethral lesion (7, 8, 13). Water—soluble contrast medium in a concentration of 20 to 30% may be used for retrograde urethrogram. This study is terminated as soon as extravasation of contrast material, with or without total loss of urethral continuity, is visualized; excessive pressure during the injection of contrast medium into the urethra may cause urethra-venous or urethra-cavernous backflow. Extravasation of contrast medium is a sign of partial or circumferential rupture. If fluoroscopy equipment is not available, this examination can be done with a portable apparatus in the emergency room (7, 8). When computed tomography equipment (CT scan) can be used, it is helpful to investigate the genitourinary tract and provides valuable information. CT scan permits excluding major injuries in the abdomen, diagnosing types of pelvic fracture and recognizing the position of the bladder after the acute trauma. A high—riding bladder is an indirect radiographic sign of posterior urethral injury, though sometimes the urethra is only stretched without rupture of its wall.

Cystographic appearance of the bladder base gives valuable information; a closed bladder neck may be indirect evidence of integrity of the proximal sphincter mechanism. Likewise, an open and funneled bladder neck should not be mistaken for associated vesical neck lesion (15). In contrast, a cystogram showing a distorted bladder neck and extravasation of contrast medium raises the suspicion of a damaged proximal sphincter (8, 13). Some authors continue to relate incontinence, severity of urethral pelvis trauma and associated bladder neck injury at the time of trauma. The importance of preserving the bladder neck sphincter function in these cases is mandatory to maintain continence after urethroplasty, as the distal sphincter mechanism at the membranous urethra is damaged at the original trauma (8, 13, 15, 16).

Traditional teaching suggests that the posterior urethra is torn off at the apex of the prostate above the urogenital diaphragm (17). However, cadaveric studies have reviewed the urogenital diaphragm anatomy and documented the non-existence of a superior layer in this diaphragm, now named perineal membrane (18). These authors encountered that the muscles surrounding the membranous urethra are connected with the muscles of the prostatic urethra, and with the perineal fascia, but not to the bulbar urethra (18). Thus, contrary to the classic anatomy description, the terminal portion of the membranous urethra, before entering the bulbar channel, is the weakest point at which the posterior urethra is exposed to traumatic rupture (19). If the rupture occurs at the distal portion of the membranous urethra, the posterior bulbar channel may be also involved (19, 20). Thus, when a violent external force causes a pelvic fracture, the pelvis is compressed and the bladder and prostate are moved upwards, stretching the membranous urethra, firmly fixed to the perineal fascia. If the trauma force exceeds the elasticity of the membranous urethra, a partial or complete rupture will take place at the membranous-bulbar urethral junction (6, 21). In cases of circumferential rupture, if the distal end of the membranous urethra remains attached to the perineal fascia the separation is less marked. Yet, if the trauma disrupts the perineal membrane the distal urethral end will be displaced to the perineum, with marked separation of the urethral extremities (21).

Colapinto and McCallum provided a classification of posterior urethral injuries: type I, posterior urethra elongated but intact; type II, partial or complete lesion of the membranous urethra above the perineal fascia and type III, partial or complete urethral tear at the bulbar-membranous junction with disruption of the urogenital diaphragm (21). In type III urethral lesion extravasation of contrast medium begins in the perineum and extends to the pelvis. In children, type III injuries seem to occur more frequently than the classic type II lesion (22). Very few authors reported pelvic fracture disruption across the prostate gland (11, 23). Boone et al. pointed out 3 distinct sites of PFPUIs in pediatric patients: suprapubic, transprostatic and prostate-membranous, while Al Rifael et al. reported ruptures across the prostate in only 3 childrens (11, 23). In the writer's experience as well as in the reports of other authors, the site of PFPUDD in children is invariably sub-prostatic (7, 12, 13). On occasions, the inframontanal prostatic urethra was also involved. These findings were confirmed by visualization of the verumontanum at the distal end of the proximal urethral extremity on preoperative antegrade urethroscopy and in the course of surgical repair (12, 13, 22). Interestingly, when concomitant bladder neck injuries were present they were longitudinal tears rather than complete transverse cuts.

Before deferred repair, pediatric patients need to be re-evaluated to define the site and length of the urethral defect, bladder neck morphology, and anatomic delineation of the anterior urethra and whether local complications such as fistulas, pseudo diverticula, or stones are present (12, 13, 15). Radiographic evaluation begins with plain radiograph of the pelvis. Combined antegrade cystography and retrograde urethrogram, as well as urethroscopy and cystourethroscopy through the suprapubic tract under anesthesia define the anatomical features of the distraction defect. We cannot overemphasize the importance of filling the prostatic urethra during cystography while retrograde urethrography is done simultaneously, to accurately delineate the length of the distraction defect (13). This difficulty is overcome with patience by repeating the cystography with different volumes of contrast medium. When the proximal urethra is not filled with contrast medium, the length of the distraction defect cannot be properly assessed.

Morey and McAninch advocated that magnetic resonance imaging (MRI) may be considered a better option to define the length and location of the urethral defect (24). Koraitim also affirmed that MRI is helpful in evaluating the anatomical features of the urethral gap and severity of prostatic displacement in 86 and 89% of 21 patients with PFPUDD, respectively (25). Limitations of MRI studies in children are that, a full bladder and a distended anterior urethra are needed with the child in a claustrophobic scenario (26).

## Results

Several questions arise in determining the management of acute traumatic posterior urethral injuries as well as in the treatment of delayed posttraumatic posterior urethral distraction defects. It is agreed that in the presence of shock, intense hemorrhage and severe associated injuries, well-judged treatment should be initially directed to stabilize the patient and treat serious simultaneous lesions, while management of the urethral lesion should be deferred. Pelvic fractures have been treated for years by conventional methods with pelvic slings or spica casts; however, nowadays, external fixation is the treatment of choice, in the presence of unstable ring fractures (27).

Early surgical exploration and suture repair have been proposed in the past, but are difficult to accomplish due to the grave condition of the patient and the limited experience of urologists managing these severe urethral injuries (17). Realignment of complete rupture of the posterior urethra in adult patients corrects lateral urethral displacement at the disrupted site, reduces the length of the distraction defect and simplifies subsequent delayed urethral repair (28). Techniques to realign the traumatized urethra differ somewhat among them; procedures can be performed either blindly or endoscopically. Endoscopic realignment is a more advantageous method, enables the urologist to identify incomplete urethral tears, which can be rapidly stented. However, in adults and particularly in children long-term reported results with this line of treatment are poor and the majority of patients require additional procedures to achieve a less than satisfactory outcome (26, 29, 30). It is agreed that in spite of these limitations, early endoscopic realignment should be reserved for pediatric patients in the following situations: (a) when urethral distraction defect is extensive, (b) when concomitant bladder neck tear is present, and (c) when associated rectal laceration requires suture (7, 13).

Nowadays, with continued refinement in surgical techniques that can treat posttraumatic urethral distraction defects with good postoperative results, low incidence of impotence and incontinence, most of the urologists prefer to place a suprapubic cystostomy followed by deferred urethral reconstruction (7, 12, 13, 23). Partial urethral disruptions may or may not result in a continuity stricture, with minimal damage of the intrinsic element of the distal sphincter mechanism. Partial tears of the urethra are managed by suprapubic cystostomy followed by successive retrograde urethrograms or urinary flow rates which will show the evolution of the lesion. If extravasation of contrast medium ceases, a micturition cystogram is performed before removing the cystostomy tube. If a stricture develops, it may respond to urethral dilatation or direct optical urethrotomy (8).

On the other hand, circumferential ruptures will almost always harm the distal sphincter mechanism and lead to a relatively short urethral distraction defect with reduced pelvic fibrosis (9, 10). Less frequently, complete ruptures may develop a long urethral distraction defect with extensive fibrous tissue in between the separated urethral ends with or without complex associated local traumatized structures (8). Suprapubic cystotomy provides effective urinary drainage without disturbing the pelvic hematoma and avoids the risk of major blood loss in a critically ill child. The main drawback is the long period during which the patient has a cystostomy before definitive surgical repair.

## Discussion

Debate is still present between those urologists who favor early urethral realignment with or without primary reconstruction of the transected urethra vs. those who advocate primary suprapubic diversion of urine and deferred repair of the urethra (17, 31). The past surgical treatment was sustained on the basis that the overcoming obliterate urethral distraction defect was not possible to treat with the surgical techniques then available. Alternatively, other authors have proposed retropubic exploration 7 to 10 days after initial trauma, when the pelvic hematoma has attained a more organized condition and the torn urethral ends can be better assessed to perform an end to end anastomosis over an indwelling catheter (8, 32). However, secondary strictures or persistent urethral distraction defects, incontinence, and erectile dysfunction were frequently found with this line of treatment (32). Moreover, in a report where primary suturing was performed, 4 of the 6 children treated in this way developed a stricture at the site of the primary anastomotic urethroplasty (15). Nerli et al. reported that 50% of their pediatric patients undergoing primary realignment needed additional endoscopic urethrotomies, while 3 of the cases required urethroplasty to manage a resultant stricture (33). Similar findings was reported in adults by Leddy et al. (34) Furthermore, in another retrospective study, no significant difference was found in the length of the established urethral gap in boys with complete PFPUI treated with early urethral realignment or suprapubic cystostomy (15). These findings are in consonance with Radge and McInnes observations during transpubic exploration of proximal urethral ruptures; these authorities found that even catheter traction did not approximate the stented ruptured urethral extremities (35).

The aim of deferred urethral reconstruction for children with PFPUDD is to restore urethral continuity with an adequate caliber and minimal life-long complications, as recurrent strictures, incontinence or erectile dysfunction. A variety of surgical procedures have been proposed for the delayed repair of PFPUDDs: urethral dilatation, endoscopic techniques which include direct optical internal urethrotomy (DVIU), substitution procedures and deferred tension-free mucosa to mucosa anastomotic repair, when the bulbar urethra is normal (13, 36).

Urethral dilatation and internal urethrotomies for PFPUDD are not acceptable in children; reported results have been poor, and patients undergoing these procedures required additional surgical operations (36, 37). DVIU has been found advantageous for both the management of annular membranous strictures following partial urethral injuries or for short non-obliterative strictures after failed post-traumatic primary anastomotic repair (38). In selected cases with minimal posterior urethral distraction defects some authors advocate the use of DVIU, which may create a passage through the dense pelvic fibrosis. However, this method is usually followed by long-term urethral dilatations and ultimately requires urethroplasty repair. Furthermore, false passages into the bladder neck or fistula development between the torn urethra and the rectum can occur with this treatment modality. Consequently, this line of treatment should be avoided in the case of obliterative membranous urethral distraction defects following pelvic fracture (37, 39).

Substitution procedures for PFPUDDs are only required when there is a specific indication: (a) simultaneous injury of the anterior urethra; (b) should the patient have a concomitant anterior urethral stricture or the presence of a congenital abnormality. In these situations the anterior urethra cannot be mobilized as a flap because retrograde blood flow along the bulbo-penile spongy tissue is impaired (8, 37). A variety of substitution procedures and their modifications have demonstrated to be reliable only during short periods of time. Clearly, no urethral substitute is as good as the urethra itself. Reported failure rate of 54% after urethra-scrotal inlay is valid evidence of its poor effectiveness in the treatment of PFPUDDs (13, 37). Alternatively, some authors have described a two-stage urethroplasty option for patients with multiple urethral strictures or in cases with several failed previous urethroplasties.The principle of this technique consists in interposing a meshed split thickness skin graft between the transected urethral ends and the perineal skin margins. Once the graft has grown, the neo-urethra is constructed in a second stage with the non-hair bearing skin from a portion of the graft (40). Other authors advocate the use of foreskin or penile skin on a pedicled basis as a one-stage procedure (41). Finally, full-thickness skin grafts do poorly in PFPUDDs cases due to the lack of a well-vascularized recipient site for the graft.

At the present day, there is almost complete consensus that restoration of urethral continuity in children and adults with PFPUDDs by anastomotic bulbo-prostatic repair is the gold standard procedure, provided the anterior urethra is healthy (7, 8, 13, 42). Success in anastomotic urethroplasty is dependent on adequate surgical exposure, excision of all fibrous tissue occupying the distraction defect, mobilization of the normal bulbar urethra, fixation of healthy mucosa at the edges of the bulbar and prostatic urethral ends and performing a tension-free spatulated anastomosis, when appropriate blood supply is present through the urethra (43, 44). A variety of surgical access options are available to perform the anastomotic repair: (a) perineal approach, (b) elaborated 1-stage perineal access, (c) transpubic (partial or total) approach, (d) progressive perineal-abdominal (transpubic) approach, and (e) posterior sagittal access (8, 42, 45, 46).

It is interesting to try to understand why some urologists prefer either the transpubic approach or the perineal access alone to restore urethral continuity in children with PFPUDDs (42, 45). One reason why these urologists make such a decision may be related to the preference of preoperative radiographic evaluation of the urethral distraction defect, rather than to the validation of the anatomical features of the traumatized urethra in the course of surgery. A priori, it could be argued that judging the characteristics of the urethral distraction defect only on the basis of preoperative imaging studies may be misleading; thus, under this scenario the perineal approach alone may result insufficient to resolve a “complex” distraction defect (8, 13, 22). Hence, the perineal access needs to be extended to achieve a wider exposure, in order to perform a tension-free spatulated anastomosis, which is extremely difficult if the patient is already placed in the high lithotomy position, frequently used in the perineal approach.

Two surgical procedures have been proposed for adult patients to solve this difficulty: the perineo-abdominal (transpubic) progression approach and the elaborated 1-stage perineal access (8, 45). The first procedure enables progression from a perineal to a perineal-abdominal access with or without partial pubectomy, according to the intra operative anatomical features of the urethral distraction defect and allowing supracrural re-routing of the mobilized urethra if needed (47). While the elaborated perineal technique provides stepwise maneuvers to accomplish a tension-free anastomosis: (a) mobilization of the bulbar urethra, (b) separation of the proximal corporeal bodies, (c) resection of the inferior margin of the subpubic arch, and (d) the possibility to reroute the anterior urethra around one corporeal body to shorten the course of the mobilized bulbar urethra (45). In a review of 38 articles with substantial contribution to the management of PFPUDDs, Hosseini et al reported a success rate of 82% to 95% with the perineal anastomotic repair (48). Singla et al. in 28 patients with PFPUDD whose mean age was 12 years at the time of injury, performed perineal anastomotic repair in 27 cases, with a success rate of 75%, but follow up ranged from 3 to 58 months (49). El-Sheikh et al also treated 15 children between 5 and 17 years of age with perineal anastomotic urethroplasty. Initial success rate was 80% with a mean follow-up of 25 months (50). Finally, Orabi et al managed 47 boys (mean age 9 years) by perineal anastomotic repair in 40, perineal anastomotic urethroplasty with inferior pubectomy in 3, transpubic repair in 4, and substitution urethroplasty in 3 other cases. Mean follow-up was 4.5 years (51). Three children who underwent perineal repair had a re-stricture, 1 after transpubic repair due to callus formation, and 1 after substitution repair (51).

It must be recognized that the progressive perineo-abdominal procedure has advantages over the elaborated perineal technique when treating pediatric patients: (a) provides a wider exposure, (b) facilitates restoration of urethral continuity in the presence of a high riding proximal urethral end and extensive fibrosis, and (c) allows concomitant repair of a damaged bladder neck and the treatment of traumatized adjacent structures. Furthermore, in the hands of the writers the elaborated perineal technique, when applied to prepubertal boys, did not allowed intercrural space development and, consequently, inferior pubectomy.

Furthermore, other authors have shown that anastomotic urethroplasty performed through perineal-abdominal transpubic access in children with PFPUDD have a success rates >90% (7, 13). Interestingly, Al- Rifaei et al. treated 20 children (2 to 18 years old) with PFPUDD. The level of the rupture was at the membranous urethra in 17 cases, across the prostate in 3 and with complete obliteration of the entire prostatic urethra in one. The approach used was perineal in 4, transpubic-abdomino- perineal anastomotic in 16. In 1 of these latter patients, a distally based anterior bladder tube was performed. Good postoperative results were noted in all perineal repairs, but 2 cases treated transpubically had recurrent strictures and 4 developed incontinence. All incontinent cases had preoperative damage of the proximal sphincter mechanism (23).

Four years later, Kardar et al treated 12 boys (3–12 years)with bulbo-prostatic anastomosis. All but one patient underwent a perineal-abdominal transpubic approach (partial pubectomy in 7 and total in 4). After mean follow-up of 22 months, there were no recurrent strictures and 8 boys were continent. Erections were noted before and after anastomotic repair in 66% of these cases (12). Likewise, Das et al reported 100% success after using transpubic urethroplasty in 10 children, all of whom are continent (52). On the basis of this line of treatment, Patil followed 5 of 30 patients 9 to13 years old, who had been treated transpubically into adulthood. All were unimpeded in daily activities, sports and sexual function (53).

Ultimately, three articles with an important number of patients and long follow-up, referred to the outcomes of the perineal and combined perineal- abdominal transpubic anastomotic repair. In the first study, Koraitim reported excellent results in 69 boys (3–15 years) with either transperineal (93%) or transpubic anastomotic repair (91%), as opposed to a failure rate of 54% with urethroscrtal inlay procedure (13). This author used the perineal approach in 42 patients when the urethral gap was up to 3 cm and transpubic repair in 23 cases with longer defects (13). In another study, risk factors that may complicate a satisfactory anastomotic urethroplasty outcome were analyzed, comparing 15 boys with PFPUDD treated with perineal bulbo-prostatic anastomosis vs. a similar number of cases managed with the perineal-abdominal transpubic anastomosis repair (22). Median follow-up of these cases was 8.5 years. Stricture-free rate in patients managed only through the perineum was 84 vs. 100% in those treated with combined (transpubic) access. Retrospectively, failed perineal urethroplasty was attributed to improper patient selection as all cases had distraction defects of at least 3 cm of length with significant cephalic displacement of the prostate. These 4 failed patients were stricture free after a combined perineal- abdominal (transpubic) anastomotic repair (3) and optical urethrotomy (1). Incontinence developed in 1 boy in the perineal group, and in 3 in the combined (tranpubic) approach, attributed to the violence of initial trauma and concomitant bladder neck tears. This study highlighted that in children with PFPUDD, surgical repair should begin through a perineum exposure and when tension–free anastomosis was not possible to perform through this access an abdominal (partial pubectomy) approach is required for the resolution of the distraction defect (22).

A more recent study reviewed 49 male children and adolescents aged 3.5 to 17.5 years (median 9.5) with PFPUDDs who underwent delayed bulbo-prostatic anastomosis. Median urethral gap defect was 3 cm (range 2–6). Access was perineal in 28 and perineal/partial pubectomy in 29. Median follow-up was 6.5 years (range 5–22) (7). Five perineal anastomotic repair cases developed recurrent strictures at the anastomosis site, successfully managed with additional perineal/partial pubectomy anastomosis (4) and internal urethrotomy (1). Primary and overall success rate was 89, 7 and 100%, respectively. Urinary incontinence occurred in 9 cases: 2 had overflow incontinence and performed self- catheterization,1 developed sphincter incontinence and required AUS placement, while 4 of 6 cases with mild stress incontinence achieved dryness at pubertal age. Retrospectively, associated bladder neck lesions at trauma time were noted in 5 of these patients. Three patients with erectile dysfunction before urethral reconstruction remained with erectile dysfunction (7).

Finally, a 9 year old patient with a PFPUDD was treated with anastomotic urethroplasty via an anterior sagittal approach without splitting the rectum with a good post-operative result though follow-up was only 18 months (54). The author reported “an excellent exposure of the posterior urethra.”

Postoperative follow-up involved clinical visits and retrograde urethrogram 1 month after repair, repeated at 1, 5, 10, and 15 years thereafter. Uroflowmetry was indicated yearly. Patients with incontinence after urethroplasty underwent video-urodynamic studies. Erectile dysfunction was identified in older children when they were able to inform of this disability (7).

It is noteworthy to admit limitations of this review. The retrospective nature of this study has the inherent flaws associated with this review design. Moreover, the exact mechanism of erectile dysfunction was not investigated in the manuscript reviewed, in the kind of detail required because of patients' age.

## Conclusions

In children, urethral injury, though uncommon, is an important cause of morbidity. Initial treatment of posttraumatic posterior urethral injuries caused by pelvic fractures should be directed to stabilize the patient and treat life-threatening associated injuries. The following step is to diagnose the urethral lesion by retrograde urethrography; if present, suprapubic urine drainage should be performed in the majority cases. Immediate realignment procedures are only required in children with PFPUI associated with concomitant bladder neck tear, simultaneous rectal laceration or when urethral distraction defect is extensive. Timing for deferred repair is postponed until local healing is complete and the hematoma has contracted, generally 3–4 months after original trauma. Preoperative assessment of the established urethral distraction defect includes combined radiographic studies and urethroscopy findings in order to define the anatomical features of the urethral distracting defect. However, precise delineation of the PFPUDD is more accurately determined at the time of surgical reconstruction. As evidenced in this review, when a healthy anterior urethra is present, resection of the pelvic fibrosis and end to end spatulated anastomosis is the gold standard technique to treat PFPUDDs. The procedure should be initiated through the perineum, only to be extended to lower abdomen, with or without a partial transpubic access, when long distraction defects and complex associations, such as simultaneous bladder neck lesions, recto-urethral fistulas or periurethral cavities are present. Substitution urethroplasty for PFPUDD are rarely necessary, reserved for cases with associated anterior urethral strictures, congenital abnormalities or in cases with several failed previous urethroplasties.

## Ethics Statement

Approval was granted by the hospital review board.

## Author Contributions

All authors listed have made a substantial, direct and intellectual contribution to the work, and approved it for publication.

### Conflict of Interest Statement

The authors declare that the research was conducted in the absence of any commercial or financial relationships that could be construed as a potential conflict of interest.

## Abbreviations

PFPUIs: pelvic fracture posterior urethral injuries
PFPUDDs: pelvic fracture posterior urethral distraction defects.
